# Supplementary figures and images for: Quantifying the role of genome size and repeat content in adaptive variation and the architecture of flowering time in Amaranthus tuberculatus
Source: PLoS Genet. 2023 Dec 27;19(12):e1010865. doi: 10.1371/journal.pgen.1010865 (PMC10775983; doi:10.1371/journal.pgen.1010865)

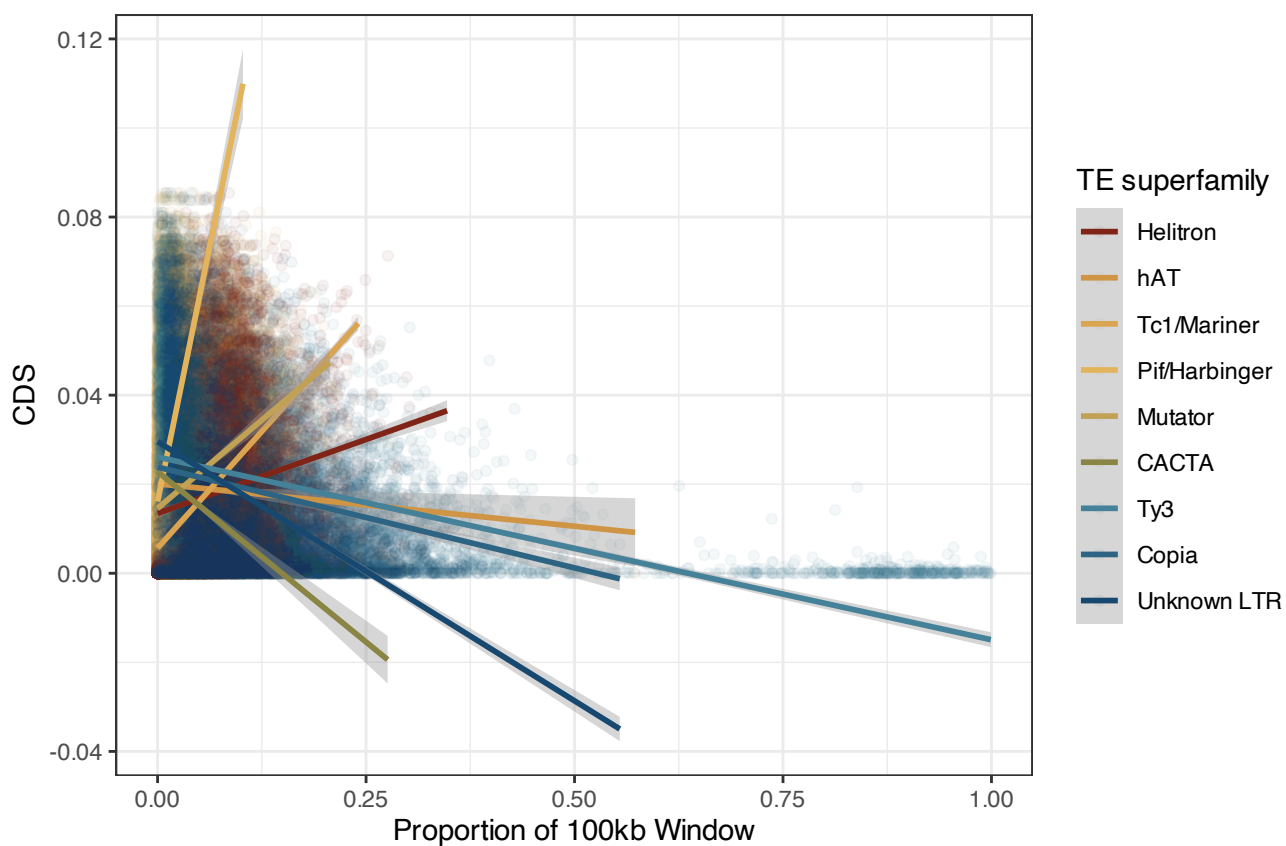

Supplement: S1 Fig — (PDF) [file pgen.1010865.s001.pdf]

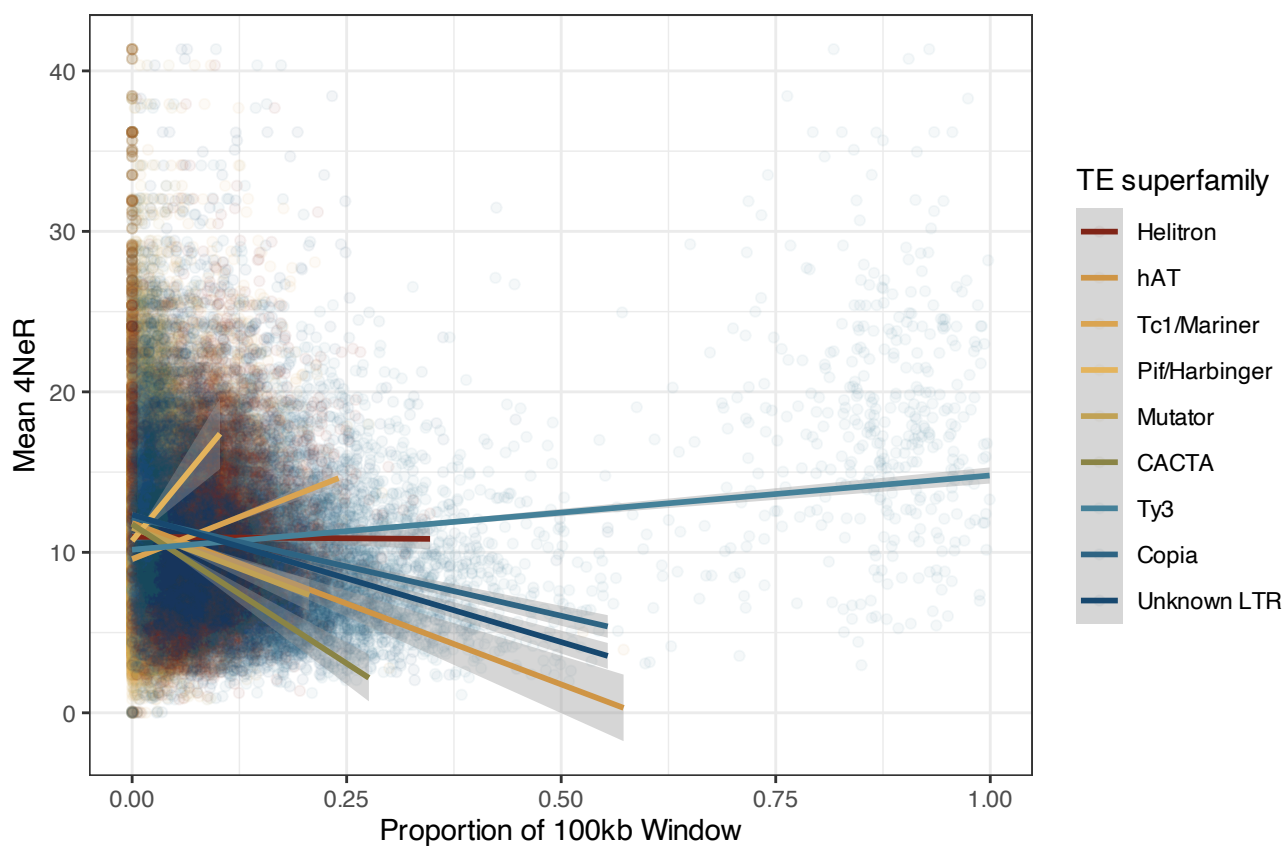

Supplement: S2 Fig — (PDF) [file pgen.1010865.s002.pdf]

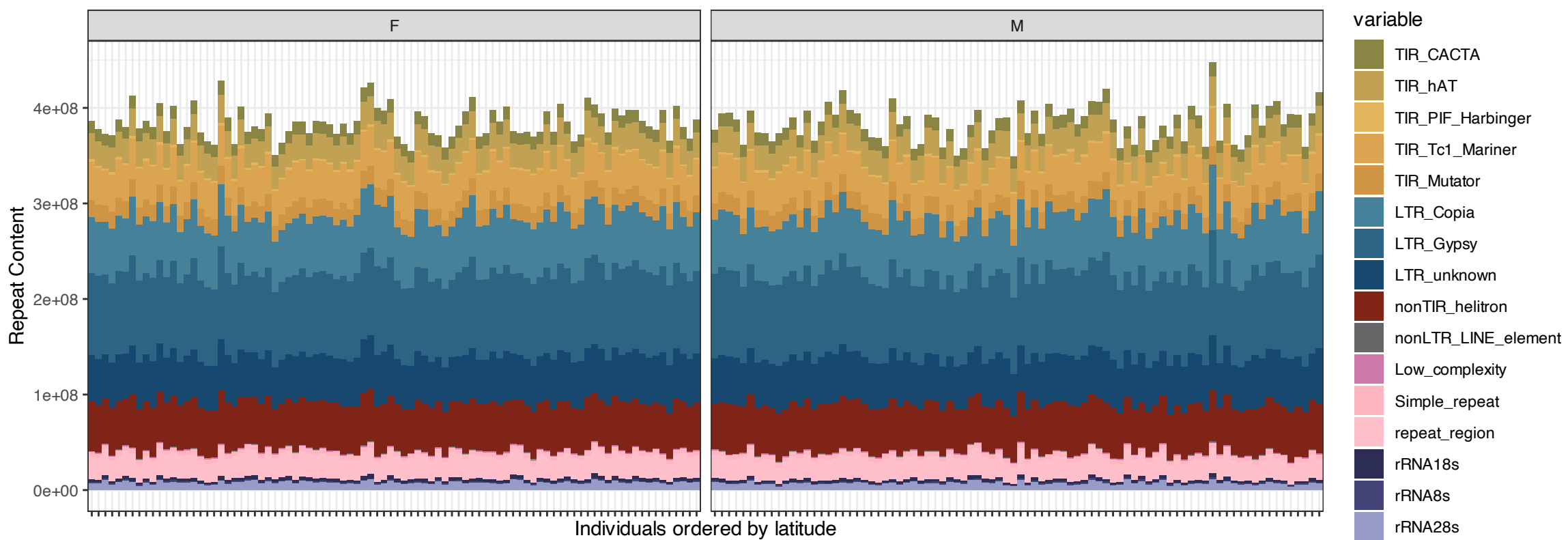

Supplement: S3 Fig — (PDF) [file pgen.1010865.s003.pdf]

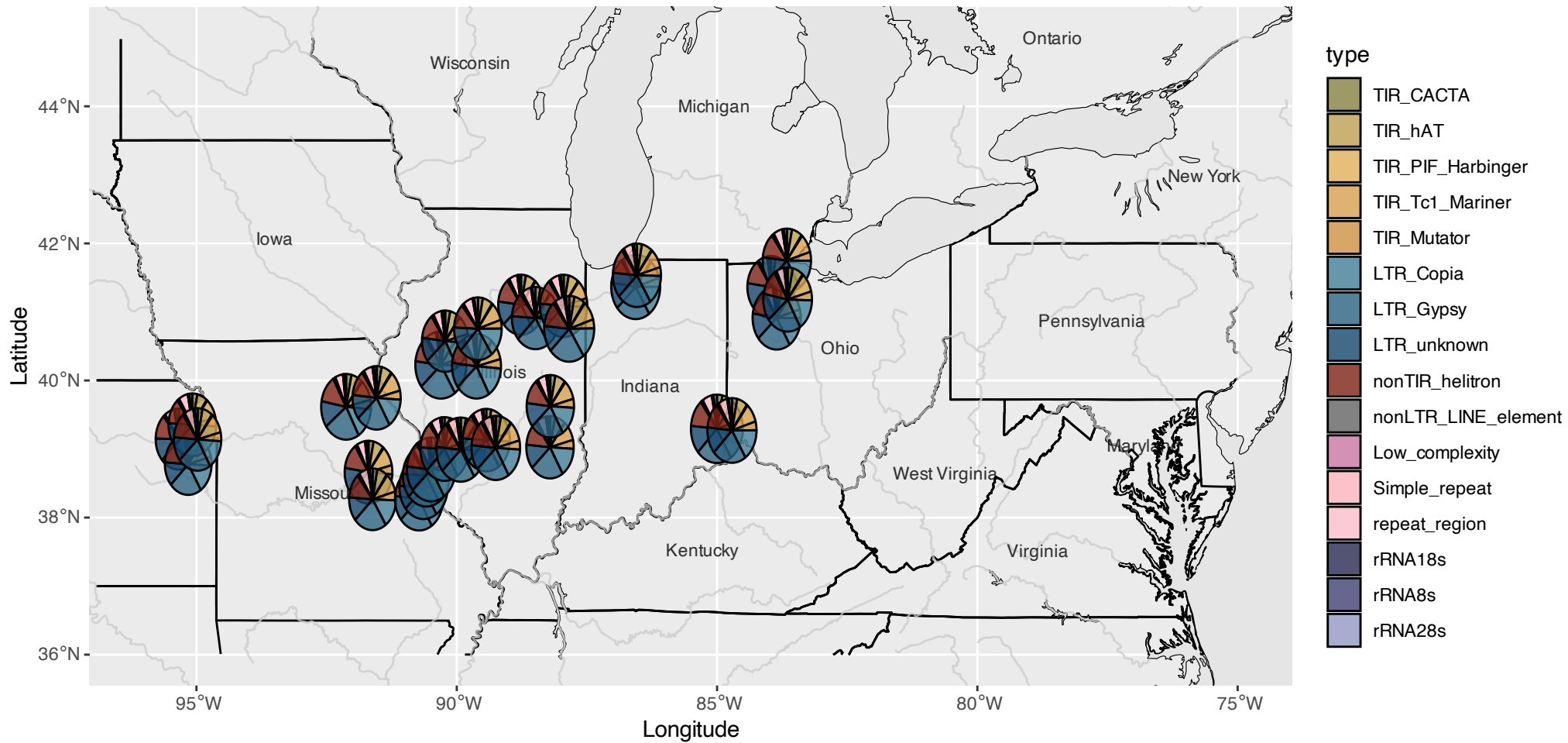

Supplement: S4 Fig — (PDF) [file pgen.1010865.s004.pdf]

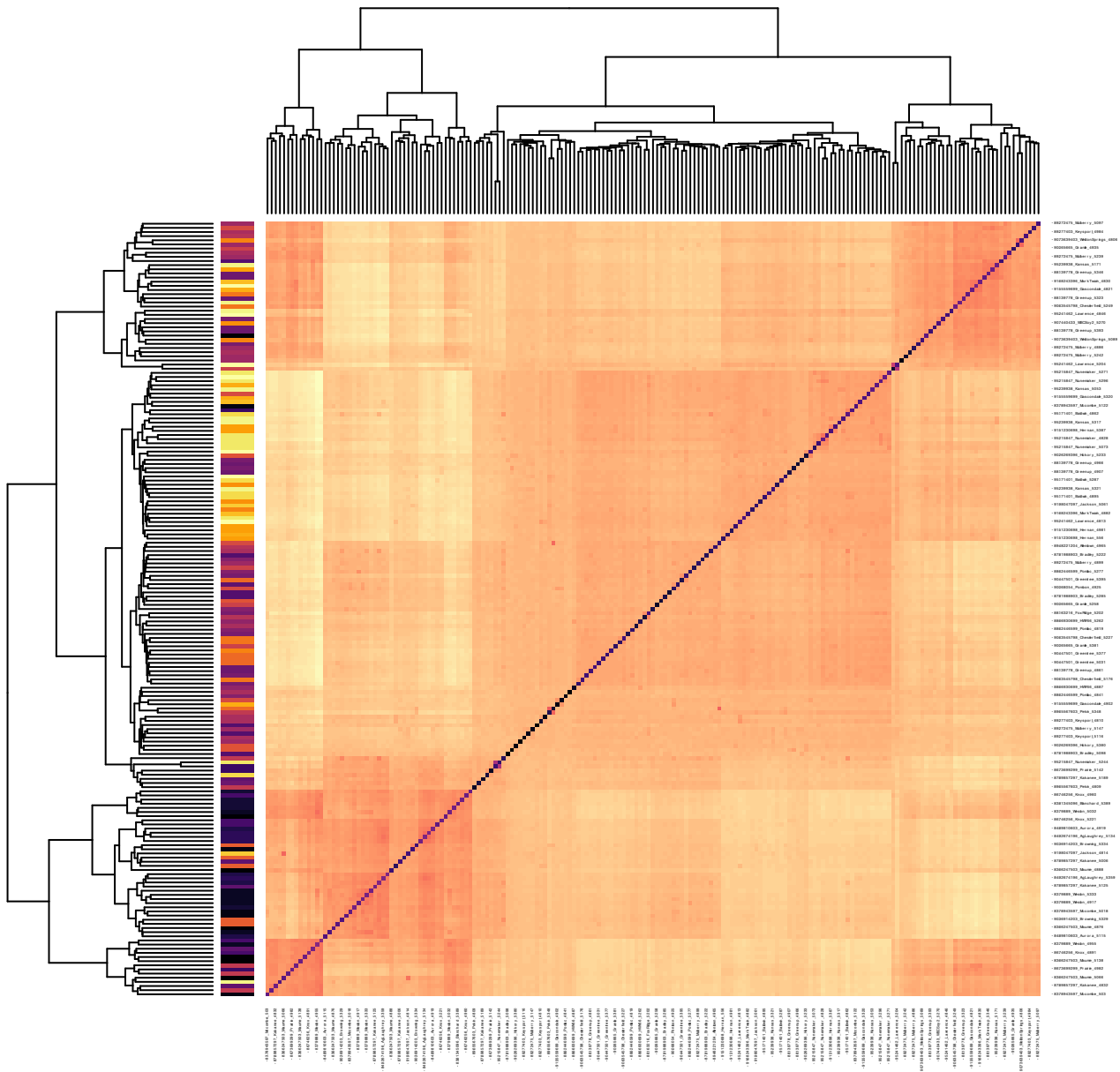

Supplement: S5 Fig — Colors on the left represent population groupings ordered by longitude, with the most eastern populations in darker colours. (PDF) [file pgen.1010865.s005.pdf]

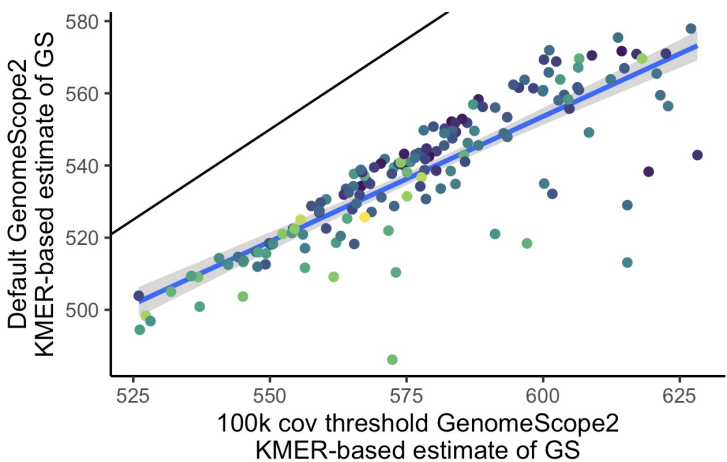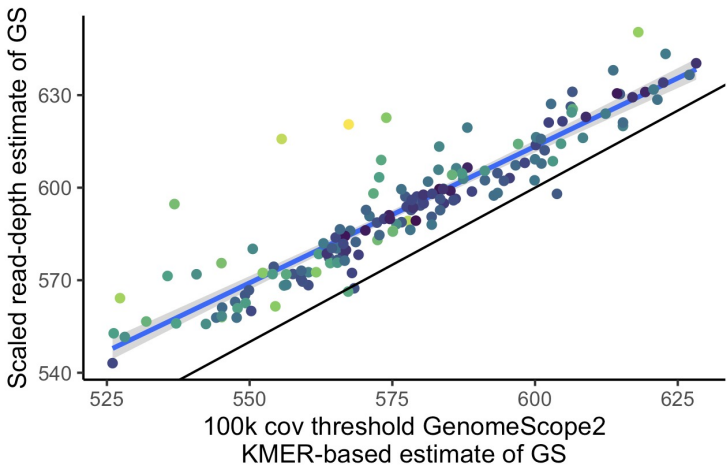

Supplement: S6 Fig — Top figure shows the relationship between kmer estimates of genome size depending on kmer coverage threshold (default = 10,000 versus 100,000). Solid black line represents the 1:1 expectation. (PDF) [file pgen.1010865.s006.pdf]

Frequency

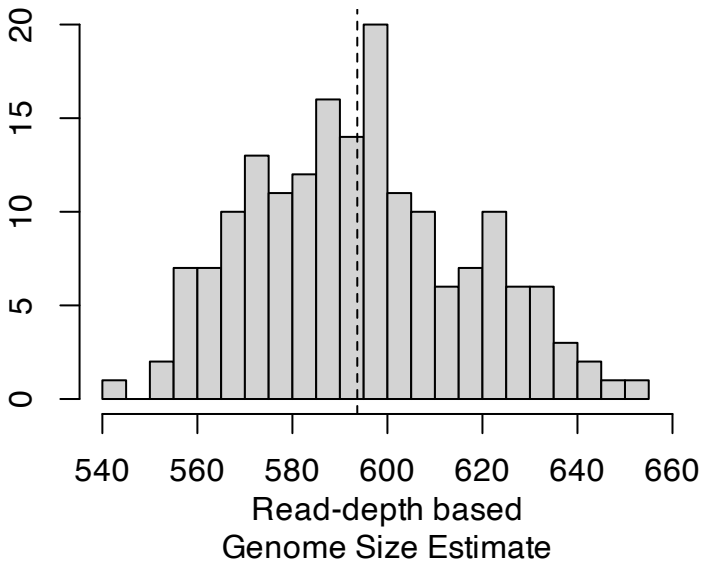

Supplement: S7 Fig — (PDF) [file pgen.1010865.s007.pdf]

**A**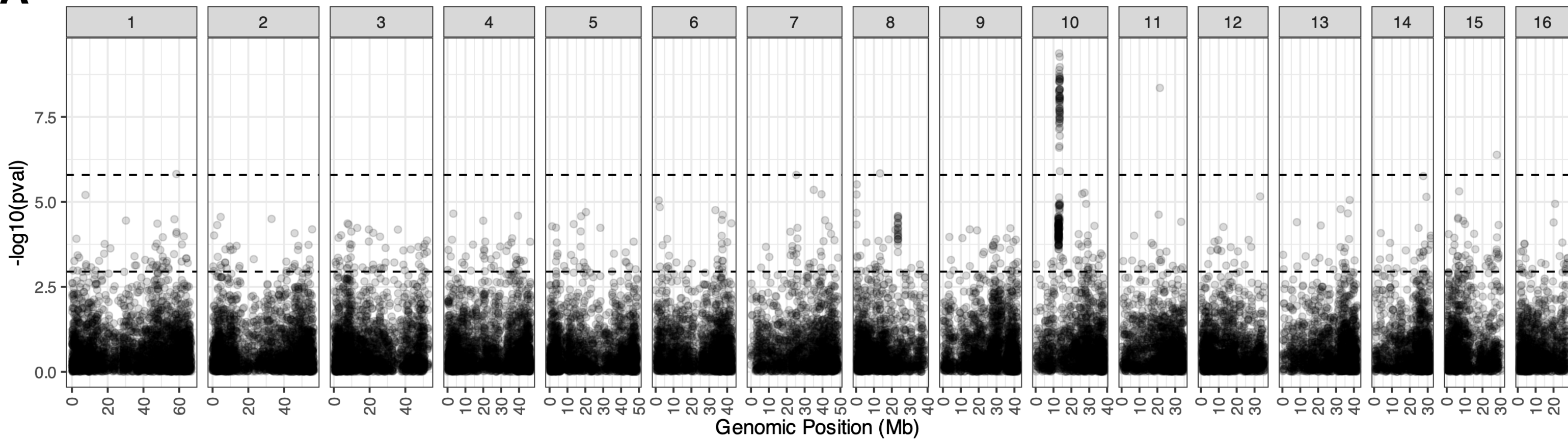**B**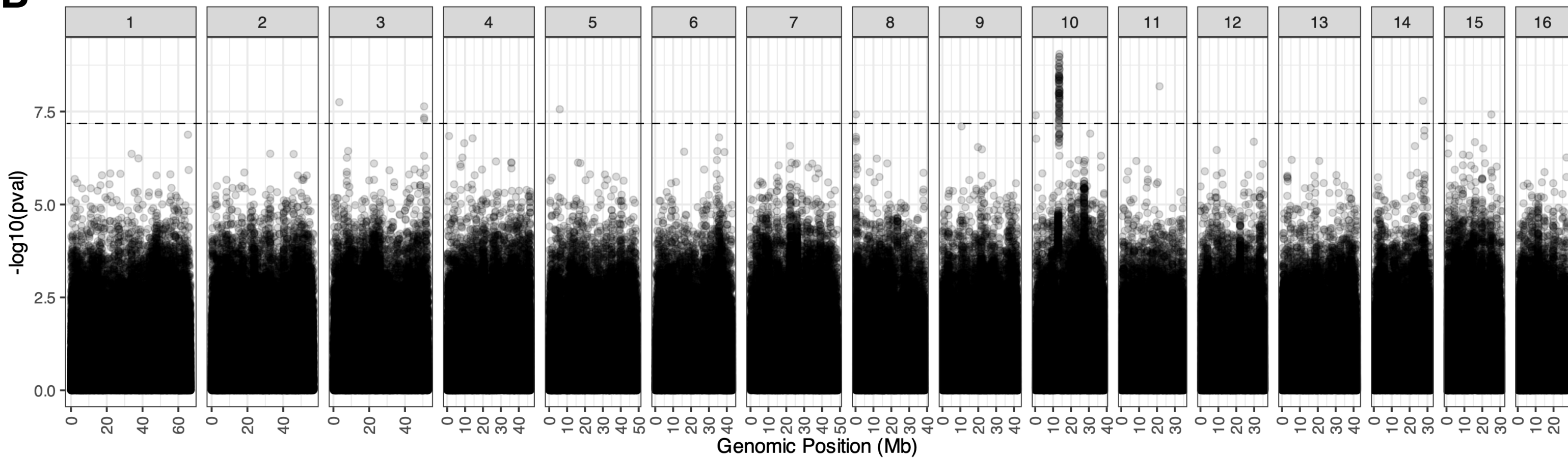

Supplement: S9 Fig — Horizontal dashed line indicates a 5% false discovery rate threshold. (PDF) [file pgen.1010865.s009.pdf]
