# Supplementary material for: Quantifying the role of genome size and repeat content in adaptive variation and the architecture of flowering time in Amaranthus tuberculatus
Source: PLoS Genet. 2023 Dec 27;19(12):e1010865. doi: 10.1371/journal.pgen.1010865 (PMC10775983; doi:10.1371/journal.pgen.1010865)
Supplement: S8 Fig — Horizontal line represents a FDR 10% cutoff. SNPs above this line are enriched for GO biological processes listed above. (PDF) [file pgen.1010865.s008.pdf]

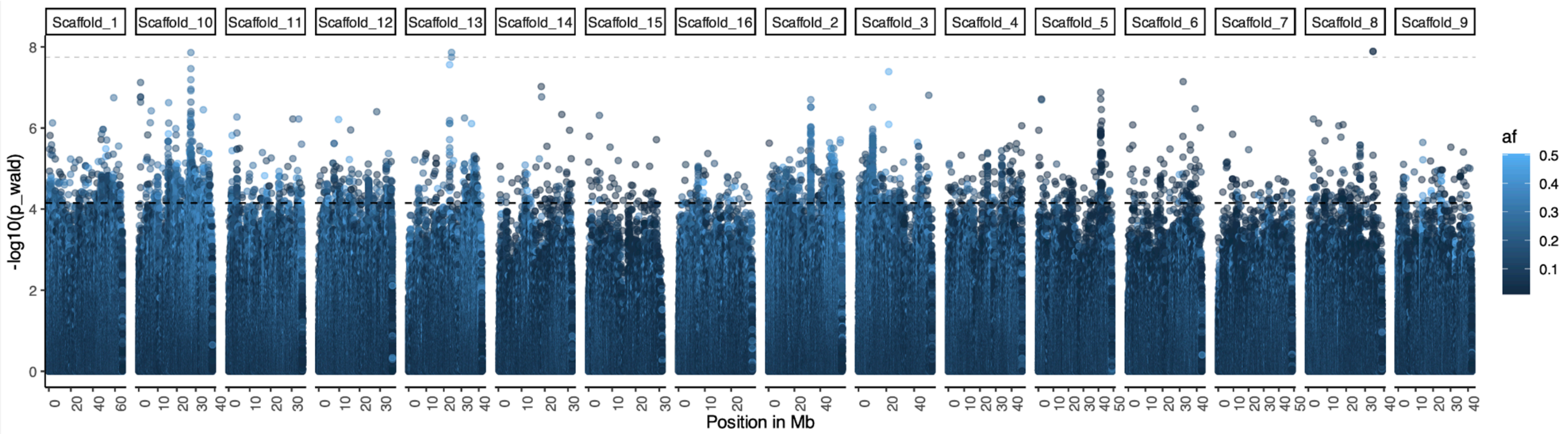

	Arabidopsis thaliana (REF)	upload_1 (▼ Hierarchy NEW! ?)					
GO biological process complete	#	#	expected	Fold Enrichment	+/-	raw P value	FDR
post-embryonic plant morphogenesis	222	19	6.56	2.90	+	7.59E-05	3.08E-02
↳anatomical structure development	5750	217	169.97	1.28	+	1.00E-04	3.79E-02
reproductive shoot system development	576	37	17.03	2.17	+	2.37E-05	1.04E-02
↳multicellular organism development	4693	193	138.72	1.39	+	1.82E-06	1.29E-03
↳multicellular organismal process	5038	202	148.92	1.36	+	5.34E-06	3.03E-03
↳system development	4000	166	118.24	1.40	+	9.10E-06	4.69E-03
↳shoot system development	1582	82	46.76	1.75	+	2.17E-06	1.37E-03
macromolecule modification	3897	156	115.19	1.35	+	1.08E-04	3.82E-02
↳macromolecule metabolic process	8518	339	251.79	1.35	+	2.53E-10	3.59E-07
↳organic substance metabolic process	12068	449	356.73	1.26	+	1.86E-10	3.52E-07
↳metabolic process	12881	489	380.76	1.28	+	6.58E-14	1.87E-10
cellular metabolic process	9211	355	272.27	1.30	+	3.02E-09	3.43E-06
↳cellular process	15242	563	450.55	1.25	+	1.97E-15	1.12E-11
primary metabolic process	9981	377	295.04	1.28	+	7.20E-09	6.81E-06
organonitrogen compound metabolic process	6355	236	187.85	1.26	+	1.23E-04	4.11E-02
↳nitrogen compound metabolic process	9043	345	267.31	1.29	+	2.44E-08	1.98E-05
response to stimulus	9743	350	288.00	1.22	+	1.11E-05	5.23E-03
